# The Gram-Negative Bacilli Isolated from Caves—*Sphingomonas paucimobilis* and *Hafnia alvei* and a Review of Their Involvement in Human Infections

**DOI:** 10.3390/ijerph19042324

**Published:** 2022-02-17

**Authors:** Mihaela Ileana Ionescu, Dan Ștefan Neagoe, Alexandra Marioara Crăciun, Oana Teodora Moldovan

**Affiliations:** 1 Iuliu Hațieganu University of Medicine and Pharmacy, 6 Louis Pasteur, 400349 Cluj-Napoca, Romania; acraciun@umfcluj.ro; 2 Department of Microbiology, County Emergency Clinical Hospital, 400006 Cluj-Napoca, Romania; danstneagoe@yahoo.com; 3 Emil Racovita Institute of Speleology, Cluj-Napoca Department, Clinicilor 5, 400006 Cluj-Napoca, Romania; oanamol35@gmail.com; 4 Romanian Institute of Science and Technology, Saturn 24-26, 400504 Cluj-Napoca, Romania

**Keywords:** *Sphingomonas paucimobilis*, *Hafnia alvei*, cave environment, bloodstream infections, urinary tract infections, pediatric infections, opportunistic infections, colistin resistance, innate antibiotic resistance, identification methods

## Abstract

The opportunistic infections with Gram-negative bacilli are frequently reported. The clinical studies are focused on the course of human infectious and very often the source of infection remain unclear. We aim to see if the Gram-negative bacilli isolated from a non-contaminated environment—the caves—are reported in human infections. Eleven samples were collected from six Romanian caves. We used the standard procedure used in our clinical laboratory for bacterial identification and for antibiotic susceptibility testing of the cave isolates. Out of the 14 bacterial strains, three isolates are Gram-negative bacilli—one isolate belong to *Hafnia alvei* and two strains belong to *Sphingomonas paucimobilis*. We screened for the published studies—full-text original articles or review articles—that reported human infections with *S. paucimobilis* and *H. alvei.* Data sources—PubMed and Cochrane library. We retrieved 447 cases from 49 references—262 cases (58.61%) are *S. paucimobilis* infections and 185 cases (41.39%) are *H. alvei* infections. The types of infections are diverse but there are some infections more frequent; there are 116 cases (44.27%) and many infections of the bloodstream with *S. paucimobilius* (116 cases) and 121 cases (65.41%) are urinary tract infections with *H. alvei*. The acquired source of the bloodstream infections is reported for 93 of *S. paucimobilis* bloodstream infections—50 cases (43%) are hospital-acquired, and 40 cases (37%) are community-acquired. Most of the infections are reported in patients with different underlying conditions. There are 80 cases (17.9%) are reported of previously healthy persons. Out of the 72 cases of pediatric infections, 62 cases (86.11%) are caused by *S. paucimobilis*. There are ten death casualties—three are *H. alvei* infections, and seven are *S. paucimobilis* infections.

## 1. Introduction

Opportunistic infections are challenging issues [1,2]. The infections with multi-drug resistant strains are spreading in the hospital environment and cause serious diseases, mainly in immune-compromised persons [3,4]. It is difficult to establish the source of opportunistic infection, mainly in the hospital environment [5]. The need for the discovery of new antibiotics has galvanized researchers to focus on microorganisms that resist antibiotics [6]. A recent report of the complete genome of the *Hafnia alvei* A23BA isolated from plant rhizosphere demonstrates that environmental strains could advance the discovery of antibiotic-producing environmental strains [7]. 

In addition, the complete genome sequence of *S. paucimobilis* strain Kira was recently published. The strain consists of 3,917,410 bp, with a G + C content of 65.7% [8]. The organisms have to adapt to a large panel of factors that are not constant—temperature, carbon sources, pollutants, etc. [9,10]. The cave environment is unique for its lack of light and constant climate, and where the contaminations with pollutants can be minimal in its deepest parts [11,12,13,14]. As opposed to the relatively pristine cave environment, hospital environments are highly contaminated with and antibiotics. In addition, the specific cleaning procedures in hospitals greatly influence the microbial community [15]. Antibiotic resistance is the most striking phenotypic feature that evolved in hospital environment. In a balanced environment, the microorganisms are in a dynamic and complex process of adaptation. The innate antibiotic resistance is an important aspect of the competition between the microorganisms and is of great interest because some antibiotics, such as colistin, are the last resort for the treatment of multi-drug resistant bacterial infections [16,17,18,19]. 

In a complex project where different aspects of caves ecosystems have been analyzed, the presence of bacterial species involved in human infections was of interest. This review aimed to evaluate the potential Gram-negative bacilli implicated in human infections from non-polluted cave environments. Gram-negative environmental bacilli could cause opportunistic infections [20,21,22]. We isolated two Gram-negative opportunistic species, *Hafnia alvei* (*H. alvei*) and *Sphingomonas paucimobilis* (*S. paucimobilis*), in samples collected from six caves. 

*H. alvei* is a Gram-negative rod that belongs to Enterobacterales and until 1978 it was placed in the genus *Enterobacter*—*Enterobacter alvei* and *Enterobacter hafniae* [23]. A brewery variety of *H. alvei* biogroup 1 is *Obesumbacterium proteus* [24]. *H. alvei* habits the intestine of humans and other animals and is found in sewage, soil, water, and dairy products. *H. alvei* is considered a potential pathogen in patients with underlying diseases [25,26]. 

*S. paucimobilis* is a strictly aerobic, non-fermentative Gram-negative rod that belongs to the genus *Pseudomonas* until 1977 [27]. *S. paucimobilis* produces a yellow pigment and could be confused with flavobacteria because its mobility is difficult to demonstrate. It was isolated from the environment and solutions used for cleaning wounds [28]. 

The clinical samples with *S. paucimobilis* or *H. alvei* are no longer considered contaminated samples or an indicator of non-conformity. The *S. paucimobilis* is intrinsically resistant to the action of polymyxin antibiotics (polymyxin B and colistin) [29,30]. Some structural modifications of the lipopolysaccharide of *S. paucimobilis* may be the results of the adaptation of these species to environmental conditions [31]. 

The present study aims to connect the biological studies with the clinical reports of opportunistic infections. The biological studies are focused on the accurate identification of the microorganisms by high-standards methods like DNA sequencing by the Sanger method [32]. The clinical reports of the infections rely on clinical laboratory identification methods. Most clinical laboratories use standard identification methods. Only well-equipped microbiological laboratories from clinical facilities could routinely perform MALDI-TOF mass spectrometry (MS) [33]. 

To link biological research to clinical trials, one approach is to use the same method for microorganisms’ identification. The other approach is to confirm the species’ identification by the MALDI-TOF MS or DNA sequencing by the Sanger method [9]. The second approach is hampering the availability of the clinical isolates. In clinical laboratories, the isolates are usually not preserved for future studies. However, the confirmation of the environmental species isolated from human infections is crucial to identify the source of contamination [34]. This study emphasized the need for a connection between the fundamental research and the clinical outcomes. In the present review, we address the type of infections according to age, underlying conditions, and course of infection with *S. paucimobilis* or *H. alvei*. We sought to have comparable outcomes with the clinical studies that reported human infectious with Gram-negative bacilli isolated from our cave samples. Therefore, we used the standard techniques methods used in clinical laboratories—Gram staining, routine culture media for primary isolation, and Vitek2 Biomerieux System for identification and antibiotic susceptibility testing. We sought the treatment of the infections with *S. paucimobilis* or *H. alvei* described in the literature. The present study is an update of the case reports and the reviews that concern the human infections with two environmental Gram-negative bacilli organisms isolated from the pristine cave—*S. paucimobilis* or *H. alvei*. We made a deep analysis of the reported cases in the literature, and we highlight the importance of accurate bacterial identification in the case of human infections with opportunistic microorganisms. 

## 2. Materials and Methods

### 2.1. Cave Samples Collection and Bacterial Identification

#### 2.1.1. Cave Samples Collection

Eleven samples were collected from six Romanian caves: Topolniţa, Cloşani, Muierilor (southern Romania), Apă din Valea Leșului, Ferice (northwestern Romania), and Tăuşoare (northern Romania) during the spring of 2019. The collected samples were *Ursus spealaeus* (the extinct cave bear), bones (Muierilor Cave) and sediments on the floor from all the caves. Samples were collected in sterile Falcon vials or plastic bags, transported on ice, and kept at −60 °C in the laboratory until analysis.

#### 2.1.2. Bacteriological Identification and Antibiotic Phenotype of the Cave Isolates

We used the standard procedure used in our clinical laboratory for bacterial identification and for antibiotic susceptibility testing. 

Routine culture media were used for bacterial isolation: nutrient broth, blood agar, MacConkey agar, Chapman agar, Sabouraud agar, and Chromogenic Modified URI-COLOUR LAB-AGAR^TM^ (culture media are from BioMaxima). The microbial identification and antibiotic susceptibility testing were made by Vitek2 Biomérieux System. The Vitek2 card types for identification were: BCL, CBC, GP, and GN. Only the *S. paucimobilis* and *H. alvei* strains were subject to antimicrobial susceptibility testing using the Vitek2 card type N222. The interpretation of antimicrobial testing was made according to the Clinical and Laboratory Standards Institute (CLSI) and the European Committee on Antimicrobial Susceptibility Testing (EUCAST) guidelines for *H. alvei*. The interpretation of antimicrobial testing for *S. paucimobilis* was made according to CLSI guideline for “Other Non-*Enterobacterales*” category. the EUCAST-based therapeutic guideline does not provide interpretative standards for *S. paucimobilis*. Colistin resistance was defined as MIC >2 μg/mL (EUCAST guidelines for *H. alvei* and *Acinetobacter baumannii*)(EUCAST—Colistin Breakpoints—guidance document 2021) [35,36,37].

### 2.2. Search Strategy for Literature Review

The search was done in PubMed database (https://www.ncbi.nlm.nih.gov/pubmed/, accessed on 12 May 2021 and Cochrane Library database (http://www.cochranelibrary.com/) (accessed on 12 May 2021). The search terms were “*Sphingomonas paucimobilis*” or “*Hafnia alvei*”.

#### References Collection, Screening, and Selection

Inclusion criteria: The free full-text original articles or review articles; only English written articles; human infections.

Exclusion criteria: Conference papers; proceedings papers; comments; book chapters; non-English written articles; animal infections; the articles with no full text available 

**Figure 1:**
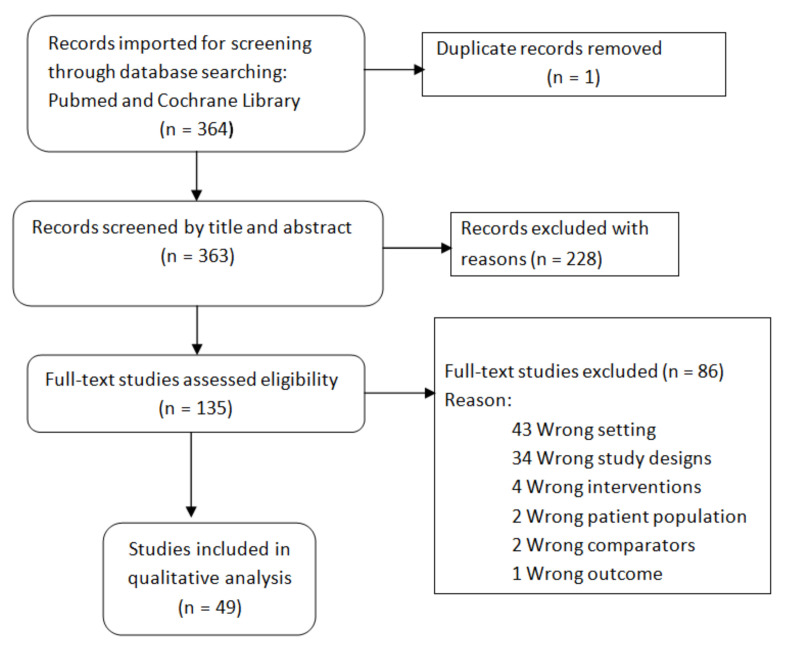
Flowchart of references selection.

The screening and evaluation for eligibility of the references retrieved were made with the Covidence software (www.covidence.org) (accessed on 12 May 2021) (Figure 1). 

### 2.3. Statistical Analysis

The results were analyzed in Excel from the Microsoft Office package. The Student’s *t*-test was used to evaluate the relationship between the samples. The *p*-value < 0.05 threshold was considered to reject the null hypothesis. Review Manager 5.4.1 was used to evaluate the forest plot. The analysis of variance (ANOVA) was used for analyses the relationship between more groups of data (*F*-value). 

## 3. Results 

### 3.1. The Cave Isolates

We collected eleven samples from six Romania caves. Fourteen bacterial strains were isolated and identified according to morphology, culture and biochemical properties. Out of the 14 bacterial strains, three were Gram-negative bacilli (Table 1). 

The present study aims to review the Gram-negative bacilli isolated from human infections. We selected the Gram-negative bacilli *S. paucimobilis* and *H. alvei*. The Gram-staining showed the Gram-negative rods, non-spore-forming. The *S. paucimobilis* produces convex, smooth, round transparent colonies, lactose-negative with yellowish pigment. On URI Chromogenic agar the *S. paucimobilis* produced small greenish-violet colonies. *S. paucimobilis* grows slowly on the nutrient broth and produces weak turbidity.

*H. alvei* organisms are small Gram-negative rods that form small, smooth, transparent, lactose-negative colonies. On URI Chromogenic agar, the *H. alvei* produced small greenish colonies. *H. alvei* produces uniform turbidity of the nutrient broth.

We selected card type N222 for antibiotic susceptibility testing of the *S. paucimobilis* and *H. alvei* strain isolated from caves. The CLSI and EUCAST MIC interpretation guidelines were compared (Table 2). For *S. paucimobilis* there are no interpretative standards in CLSI and EUCAST guidelines. We characterize the *S. paucimobilis* according to the CLSI standards for “Other Non-Enterobacterales” and according to the EUCAST standards for the non-fermentative bacilli *Acinetobacter baumannii* [35,36]. 

### 3.2. The Results of the Literature Review

#### 3.2.1. Type of Studies

**Figure 2:**
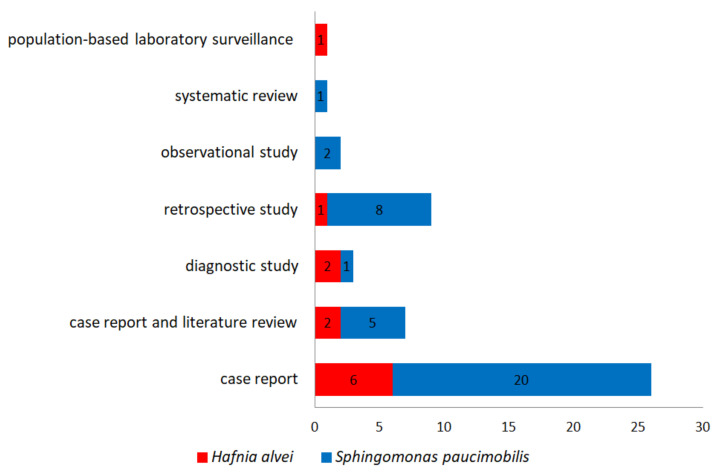
The number of the references (study types) included in the present review.

Because the type of infections with *S. paucimobilis* and *H. alvei* are rare, most of the studies are case reports. Twenty-eight of the 49 references included in this review are case reports; four are case report and literature reviews, and four are case report reviews (Figure 2).

#### 3.2.2. The Case Report and Literature Reviews

**Figure 3:**
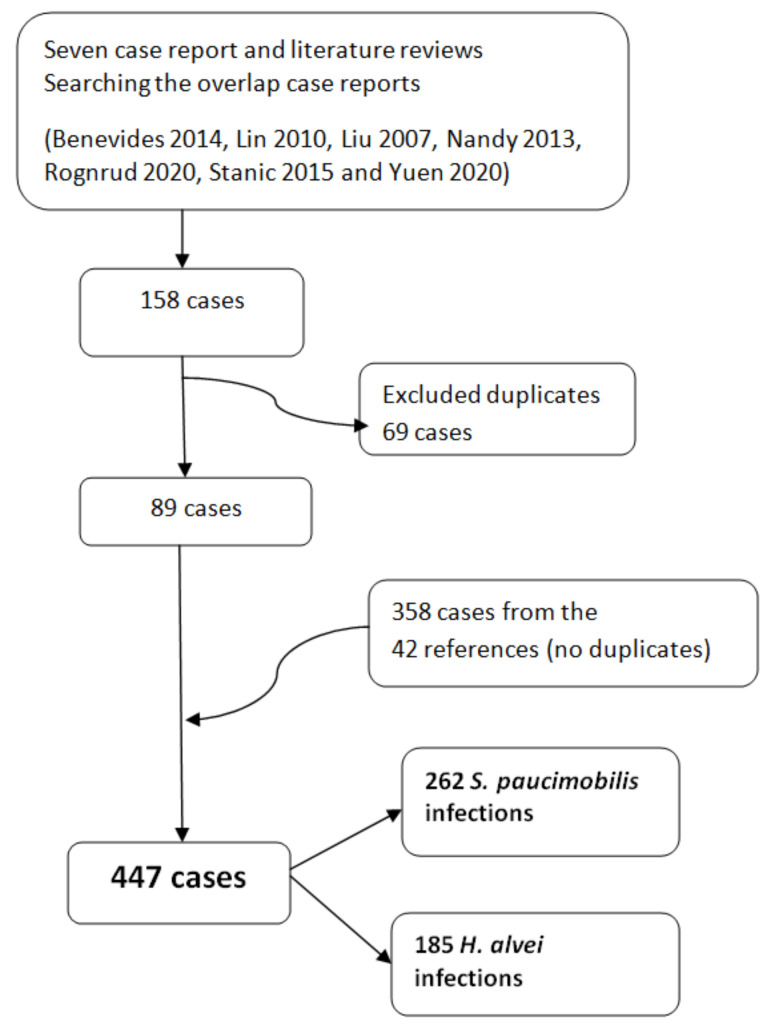
The number of case reports/infections with *S. paucimobilis* and *H. alvei* retrieved from the 49 references included in the present review. The duplicated were excluded.

There are seven references for case reports and literature reviews included in our study (Figure 2) [38,39,40,41,42,43,44]. We analyzed all the references cited in these seven references. We eliminate the duplicate case reports when count the total number of cases (Figure 3). When the literature review was not clearly systematized, we considered only the case report of the study [42]. We retrieved for further analyses 89 cases from the seven case reports and literature review references [38,39,40,41,42,43,44]. After screening the rest of the 42 references, we retrieved 358 cases. No duplicates were found. We included in our analysis 447 cases—262 *S. paucimobilis* infections and 185 *H. alvei* infections (Figure 3) [34,45,46,47,48,49,50,51,52,53,54,55,56,57,58,59,60,61,62,63,64,65,66,67,68,69,70,71,72,73,74,75,76,77,78,79,80,81,82,83,84].

#### 3.2.3. Type of Infections

The infections with *S. paucimobilis* and *H. alvei* are diverse and are reported both in immune-competent persons and in persons with underlying conditions. There are some differences in the type of infections reported with *S. paucimobilis* and compared to *H. alvei*. The most frequent infections with *S. paucimobilis* are bloodstream infections (BSI). The infections with *H. alvei* most frequently reported are urinary tract infections (UTI). The one-tailed two independent *t*-Test was made to compare the infections with *S. paucimobilis* and *H. alvei.* There was no statistical difference (*p* = 0.3) between the *S. paucimobilis* infections (*M* = 29.11, *SD* = 35.51) and *H. alvei* infections (*M* = 20.56, *SD* = 38.79) (Table 3).

**Figure 4:**
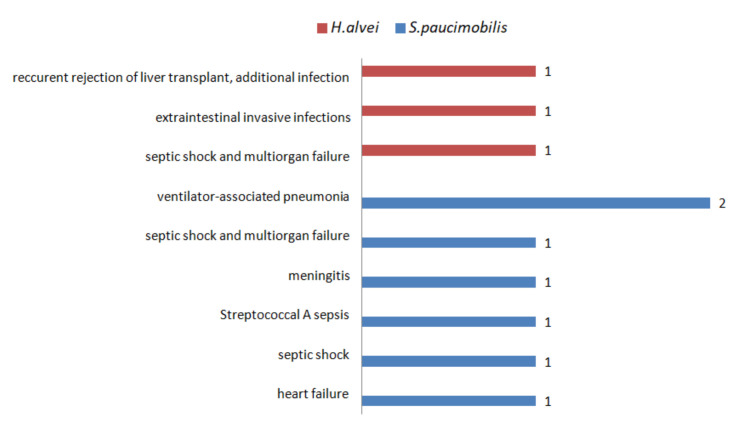
The number of cases with lethal outcomes of *S. paucimobilis* and *H. alvei* infections.

In most of the cases, the outcome of *S. paucimobilis* and *H. alvei* infections is favorable. There are ten death casualties reported despite the antibiotic treatment (Figure 4) [34,40,43,47,48,65,70,71,75]. 

#### 3.2.4. The Bloodstream Infections

**Figure 5:**
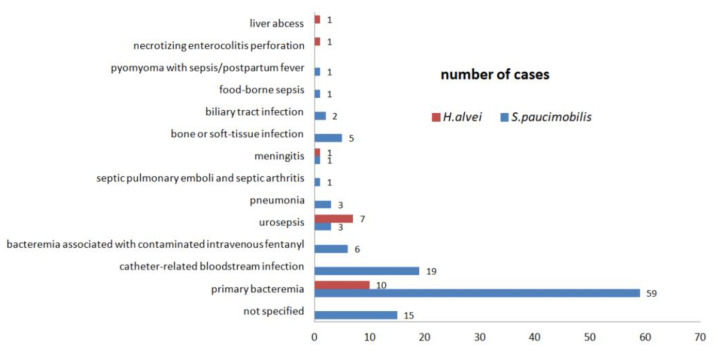
The source of infection is associated with bloodstream infections. Standard deviation *s*. *S. paucimobilis s =* 15.67; *H. alvei s* = 3.08.

**Figure 6:**
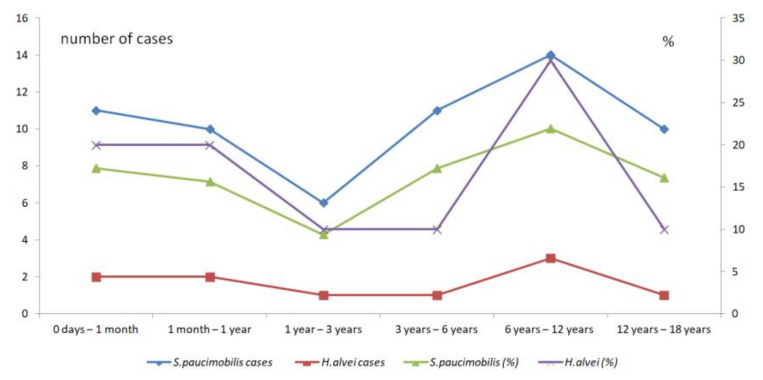
The comparison of the pediatric *S. paucimobilis* and *H. alvei* infections according to age.

Out of the 447 cases included in the present review, 136 (30.42%) are bloodstream infections (BSIs). Out of 136 BSIs, 116 (85.29%) are *S. paucimobilis* infections and 20 (14.71%) are *H. alvei* infections. We further proceed with a deeper analysis of the BSIs because the studies provide comparable details about the origin of infection, the acquired source, and the underlying conditions associated with BSIs (Table 3, Figure 5 and Figure 6).

Six references provide details about the acquired source of *S. paucimobilis* BSIs (Figure 6) [39,41,56,59,65,71]. Out of the 116 *S. paucimobilis* BSIs, 50 cases (43%) are hospital-acquired BSIs, and 43 (37%) cases are community-acquired BSIs. For the *H. alvei* BSIs, there are no details available regarding the acquired source of infection (Table 4). The acquired source of infection is an important indicator when analyzing the environmental species. The accurate bacterial identification by routine laboratory procedures is crucial in order to establish the source of infection and the treatment options. 

The underlying condition of the patients with BSIs is an important indicator when analyzing the clinical outcomes. Most of the patients have severe underlying conditions—malignancies or diabetes mellitus (Table 5).

#### 3.2.5. The Urinary Tract Infections

Out of the 125 UTIs, 121 (97.58%) are *H. alvei* UTIs (Table 3). Laupland et al., in a population-based laboratory surveillance study conducted in the Calgary Health Region during 2000–2005, show the urine was the most common focus of *H. alvei* isolation—112 (81.16%) cases from a total of 138 patients. The identification and the antibiotic susceptibility testing were performed using Vitek Biomérieux System [51]. The Rahman et al., in a retrospective study about the UTIs in female subjects, shows that the *H. alvei* are less frequent (2%) in urine samples compared with *E. coli* (69%) or other bacterial species. The bacterial identification was made by conventional biochemical tests and antibiotic susceptibility testing was performed by disk diffusion method [61]. Toh et al., in a retrospective study, reported two *S. paucimobilis* UTIs that were healthcare-associated. The identification was made with Bactec or API 20NE Biomérieux System and antibiotic susceptibility testing was performed by disk diffusion method [71]. Demir et al. and Hassan et al. reported two cases of *S. paucimobilis* UTIs reported in immune-deficient patients with multiple underlying conditions [50,83]. The sepsis combined with UTI was described in immune-deficient patients. The *H. alvei* urosepsis is more frequent than *S. paucimobilis* urosepsis (Figure 5). 

#### 3.2.6. The Respiratory Tract Infections

There are 28 cases of respiratory tract infections—17 *S. paucimobilis* infections and 11 *H. alvei* infections (Table 3). It is described a case of *S. paucimobilis* ventilator-associated pneumonia with significant dysbiosis associated COVID-19 [80]. These demonstrate the interest in clinical identification tests for environmental species that could be responsible for infections in immune-deficient patients. The decision about the presence of environmental species in a sample greatly depends on the response of the following question. Is it contamination or infection? Laupland et al., in a population-based laboratory surveillance study, reported ten (7.25%) lower respiratory infections with *H. alvei* from a total of 138 patients—seven of the lower respiratory infections were hospital infections [51]. Toh et al. reported 12 *S. paucimobilis* pneumonia/empyema infections, ten of them ventilator-associated pneumonia [71]. Apart from the lower respiratory infections reported in tertiary care units, a rare case of Yuan et al. reported a case of *S. paucimobilis* empyema secondary to foreign body aspiration [77]. Eckrich et al. reported a case of *S. paucimobilis* involvement of small airway disease in a patient with cystic fibrosis. The study includes a healthy control group [84]. 

#### 3.2.7. The Pediatric Infections

A particular characteristic of opportunistic infections is their occurrence in children. Out of the 447 cases included in the present review, 72 (16.11%) are pediatric infections. The studies concerning pediatrics offer accurate data about the age range, type of infections, and underlying conditions. We could make a detailed analysis of the 72 cases *S. paucimobilis* and *H. alvei* infections in children identified in the references included in the present review. Out of the 72 infections in children, 62 (86.11%) are *S. paucimobilis* infections. Most infections have been described in school-aged children (six years to 12 years). There is a significant difference between the pediatric *S. paucimobilis* infections (*M* = 10.33, *SD* = 2.58) compared with pediatric *H. alvei* infections according to age (*M* = 1.67, *SD* = 0.82) (Table 6). 

However, when compares the percentages of pediatric infections according to ages there is no significant difference (*t*(10) = - 0.12, *p* = 0.45) between the *S. paucimobilis* infections (*M* = 16.23, *SD* = 4.03) and *H. alvei* infections (*M* = 16.67, *SD* = 8.16). More, the peak of infections is between 6 years to 12 years for both species (Figure 6). 

The detailed comparison of the *S. paucimobilis* and *H. alvei* reveals that there is no difference between the types of infections in pediatric and adult cases. However, the panel of types of infection is more diversified in adult infections (Table 7).

The underlying conditions associated with *S. paucimobilis* and *H. alvei* infections are diverse. The most frequent underlying conditions are associated with the impairment of immunity and neutropenia, consequently of malignant diseases. We noticed the infections resulted as direct or indirect contamination—trauma, ventilatory-associated pneumonia, peritoneal dialysis-associated peritonitis, or bacteremia associated with contaminated intravenous fentanyl. There are infections in patients with no underlying conditions—in our study, we identified 80 (17.9%) cases reported in previously healthy patients (Table 8). However, in many cases, the source of infection remains elusive. This is an important indicator that could advance the understanding of these types of infections. 

Malignant diseases were reported both in adult and pediatric infections with *S. paucimobilis* and *H. alvei.* However, there were notable differences about the type of malignancy when there were details about the nature of the malignancy.

In adult infections, the most frequent are the solid cancers—oral cancer, hepatocellular carcinoma, colon cancer, hypopharyngeal cancer, esophageal cancer, bladder carcinoma, breast cancer, cholangiocarcinoma, and ovarian cancer. 

In pediatric infections, the most frequent are the blood and bone marrow cancers—acute lymphoblastic leukemia, aplastic anemia, lymphoma, non-Hodgkin’s lymphoma, acute myeloid leukemia, and acute non-lymphocytic leukemia after allogenic bone marrow transplantation. In addition, the solid cancers associated with pediatric infections are different from those reported in adult infections—neuroblastoma, anaplastic ependymoma, localized osteosarcoma, and Ewing sarcoma. 

The types of infections are various and not always explained by underlying conditions. Out of 72 of the pediatric cases, 27 (37.5%) involved children with no underlying conditions. In this respect, there are open questions about the *S. paucimobilis* and *H. alvei* infections in children regarding the infections with environmental species in healthy children. What could other host factors be responsible for the initiation and evolution of these infections? Out of the 72 pediatric cases, 23 (31.94%) were reported in children with malignancy—one case is *H. alvei* infection, and 22 cases were *S. paucimobilis* infections (Table 4). 

#### 3.2.8. The Studies with Healthy-Control Groups

**Figure 7:**
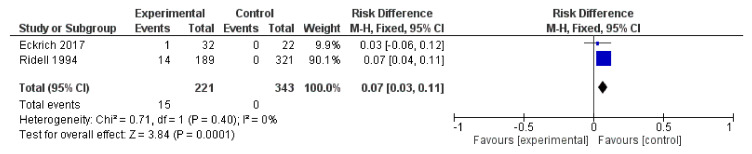
The comparison of health-control groups with the *S. paucimobilis* small airway disease (Eckrick et al.) and *H. alvei* diarrhea (Ridell et al.), respectively.

There are two studies that enrolled healthy-control groups—the Eckrich et al. study involving small airway disease in mild cystic fibrosis and the Ridell et al. study about the association of *H. alvei* with diarrhea [63,84]. Eckrich et al. conclude that the sputum neutrophils is the most informative indicator to prevent lung damage and identify *Pseudomonas aeruginosa* and *Staphylococcus aureus,* the most frequent species that colonize the airways in cystic fibrosis. Out of 32 cystic fibrosis cases, one case is *S. paucimobilis* infection [84]. The Ridell et al. study stressed that the *H.alvei* involvement in diarrhea is due to a mechanism that differs from the attachment–effacement mechanism [63]. We selected the *S. paucimobilis* and *H. alvei* cases and in a forest plot, we observed the probability of a healthy person to be infected with these two bacterial species (Figure 7). Because of the limited number of studies with healthy-control groups available, the interpretation of the results is not accurate. 

#### 3.2.9. The Microbiological Diagnostic Methods

**Figure 8:**
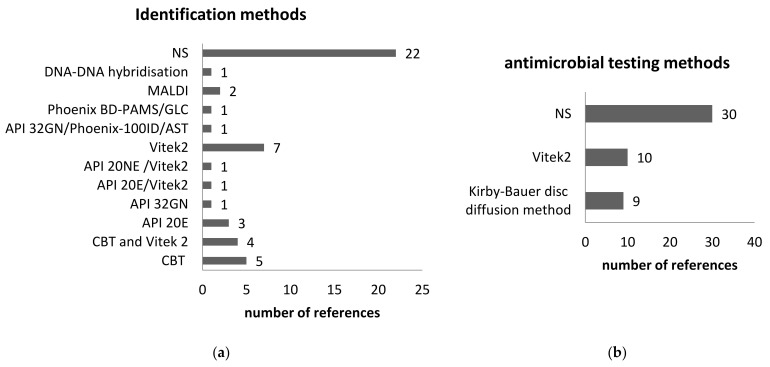
The bacteriological diagnostic was reported on the 49 references included in the study. (**a**) Identification methods; (**b**) antimicrobial susceptibility testing. NS—not specified, Phoenix BD-PAMS—BD Phoenix Automated Microbiology System and gas-liquid chromatography, GLC—gas-liquid chromatography; CBT- conventional biochemical tests.

The accurate bacterial species is crucial to the diagnostic process. The treatment greatly depends on laboratory findings. However, routine diagnostic tests are designed mainly for bacterial species that are often isolated from human infections. The environmental species are rarely reported—in our study, these case reports. Yet, the environmental species are isolated not only from immune-compromised persons but from healthy persons. In addition, very often the environmental species inherit the gene of antibiotic resistance. It is of great interest to accurately establish the antibiotic resistance phenotype. The laboratory diagnostic methods and antimicrobial susceptibility were specified in the references and are presented in Figure 8. When specified, the bacteriological methods mostly rely on conventional methods. 

### 3.3. The Innate or Natural Antibiotic Resistance

The environmental bacterial species are susceptible to most classes of antibiotics. The innate or natural resistance to some antibiotics is described for *S. paucimobilis* and *H. alvei*. The EUCAST guidelines reported the innate resistance of *H. alvei* and *H. paraalvei* at aminopenicillins, aminopenicillins and beta-lactamases inhibitors, cephalosporin’s first generations, and colistin. The CLSI guidelines reported the innate resistance of *H. alvei* at ampicillin, amoxicillin-clavulanic acid, ampicillin-sulbactam, cephalosporins I (cefazolin, cephalothin), and cephamycins (cefoxitin, cefotetan) [35,36]. Holmes B. et al. described the resistance of *Hafnia* isolates to cephalosporins and penicillin [24]. The CLSI and EUCAST guidelines are focused on the bacterial species of medical interest. There are no indications of the innate resistance for *S. paucimobilis*. Pitt T.L. mentioned that most of the *S. paucimobilis* are resistant to ureidopenicillins and ‘earlier’ cephalosporins [28].

The three bacterial species isolated from caves have an inherent resistance to colistin. Although the commercial cards Vitek2 for antimicrobial testing do not accurately determine the resistance at colistin, we further analyzed the results. It is well-known that *S. paucimobilis* has inherent resistance to colistin and the *H. alvei* do not have an inherent resistance to colistin. However, Jayol et al. suggest that the colistin resistance is underestimated by conventional antibiotic testing methods, and *Hafnia* is a naturally colistin-resistant enterobacterial genus [85]. 

### 3.4. The Limitations of the Study

The reports of opportunistic infections with environmental species are rare, and the characteristics are not constantly reported. 

The commercial Vitek2 identification cards are suitable for the identification of clinically significant bacterial species. However, the environmental species require slightly different growing conditions—e.g., lower temperature and a prolonged time of incubation. The actual EUCAST guidelines recommend the micro-dilution broth method for colistin, which is not a routine method in clinical laboratories. 

### 3.5. The Strength of the Study

The study links the research aspects of the environmental bacterial species with the clinical characterization of human infections. We started our study by screening a balanced environment—the caves—in order to isolate the Gram-negative bacilli that are already reported in the literature in opportunistic infections. The use of the identification method and antimicrobial testing method currently used in clinical laboratories permits an overview of human infections with environmental Gram-negative bacilli. 

## 4. Discussion

In our study, we isolated 14 bacterial strains from 11 samples collected from six Romanian caves; some of these species could cause human infections. *Aerococcus viridian* is a rarely reported Gram-positive cocci, opportunistic organism in endocarditis or urinary tract infections [86,87]. *Rhodococcus coprophilus* is a Gram-positive aerobic bacteria that is a fecal indicator of freshwater [88]. *Bacillus cereus* is a Gram-positive spore-forming rod associated with food poisoning and rarely with local and severe systemic infections [89]. *Bacillus smithii* is a thermophilic Gram-positive rod recently evaluated as a probiotic candidate in inflammatory bowel disease treatment [90]. *Geobacillus thermoleovoran*s is a recently sequenced Gram-positive thermophilic bacteria [91]. *Geobacillus* species are evaluated for applications in biotechnology [92]. *Corynebacterium afermentans* is a Gram-positive rod rarely isolated from brain and liver abscesses and orthopedic infections [93,94,95,96]. In the present study, we selected the Gram-negative bacilli reported in human infections—*H. alvei* and *S. paucimobilis*.

There are many studies attempting to respond to the question about the relevance of the presence of an environmental bacterial species in clinical samples. The lack of uniformity in reported data does not allow advancing the analysis in many aspects—the source of infections, the clinical data, the treatment, and the identification methods. Laupland et al. admitted that, in their population-based study, the *H. alvei* infections are overestimated for two potential reasons—lack of clinical data and the lack of confirmation of the residence status of the patients included in the study [51]. We identified another risk of bias—the accuracy of the identification method. The standard tests used in clinical laboratories are designed to identify the most encountered bacterial species isolated in human infections. In our study, the *H. alvei* cave isolate was identified with 86% probability according to Vitek2 Biomérieux System. 

In a recent study, Yu et al., using a 16S rRNA gene sequence analysis, demonstrated that a *Vogesella perlucida* isolate was misidentified as *H. alvei* by traditional microbiological testing [97]. The *H. alvei* isolate exhibits colistin resistance, which is unusual for an environmental isolate. Even though human infections with environmental species are rare, the present study shows that *S. paucimobilis* and *H. alvei* infections are reported in a large panel of samples both in children and adults. 

The source of infection is of great interest in terms of isolated opportunistic species. Effective treatment depends on the decision about the nature of the presence of bacterial specie isolated. However, even when the possibility of contamination is very unlikely, it is impossible to determine the source of infection [47]. Saboor et al. hypothesized that drinking water was a source of *S. paucimobilis* BSI in an immune-competent 10-year-old boy based on the ubiquity of this bacterial species [65]. 

Ventilator-associated pneumonia is very often life-threatening. In the context of the ongoing COVID-19 pandemic, Cutuli et al. reported a *H. alvei* pneumonia in patients that need mechanical ventilation. The authors stressed the importance of monitoring the microbiota to early diagnose infectious diseases [80]. A very recent case report highlighted that the rapid and accurate diagnosis of *H. alvei* infection elucidated the diagnosis of suspicious pulmonary masses [98]. The analysis of opportunistic species in their natural environment could advance the understanding of their involvement in human infections. Very often, opportunistic infections are reported in an immune-compromised host with underlying conditions. Our study revealed that in practice infections with *S. paucimobilis* and *H. alvei* are reported in immune-compromised and immune-competent patients. 

Some authors highlighted that they do not notice an apparent immune suppression [38,39,41,47,56,65]. However, because the opportunistic infections are the consequence of a lack of equilibrium with a host-microorganism, it is relevant to analyze both sides of the balance. This was the main reason for our study, from searching the Gram-negative species from samples collected from a non-contaminated habitat—the caves. The competition of the microorganisms that inhabit a specific environment is a complex and dynamic process that depends on many factors. The analysis of the factors that act in concert in a balanced environment is beyond the aim of the present paper, but the present review revealed that the infections with *S. paucimobilis* and *H. alvei* are reported worldwide and there are many open questions about the source of contamination and about the relation host-microorganism. 

The source of infections is an important indicator to distinguish between hospital-acquired infections and community-acquired infections. In the clinical reports, this indicator is reported mainly for BSIs. The samples are taken on admission from the previously hospitalized patients, but it is not always easy to determine the origin of the infection. Ryan et al. reported the BSIs due to *S. paucimobilis* in patients with an underlying disease or condition that determines an unfavorable clinical outcome [99]. 

In contrast, we identify the *S. paucimobilis* in patients with no underlying conditions reported—22 infections and one pediatric infection. Furthermore, for 76 *S. paucimobilis* infections (one adult infection and 75 pediatric infections) the presence of underlying conditions is unclear. Similarly, there were 57 *H. alvei* infections (five adult infections and 52 pediatric infections) reported from patients with no underlying conditions. There are 114 pediatric *H. alvei* infections for which the presence of underlying conditions is unclear. In our opinion, the clinical context of infections with low pathogens is crucial.

The infections with *S. paucimobilis* and *H. alvei* were successfully treated in most of the cases analyzed in the present review. In our study, we identified a few deaths reported in highly debilitated patients. 

An important issue in clinical laboratories is the accurate identification of bacterial species isolated from human infection by routine laboratory tests. Even the environmental species are considered opportunistic—our study shows that there are many case reports that highlight a large panel of infections with two environmental species—*H. alvei* and *S. paucimobilis*. In order to have comparative methods with the clinical reports, we chose to maintain routine methods for identification. However, the present study is part of a larger study where a huge number of cave isolates will be identified by sequence-based bacterial analysis, which was 16S rRNA sequencing by the Sanger method [9]. 

We are interested in the antibiotic-resistance phenotype of bacterial species isolated from uncontaminated environments. The issue is relevant in the context of the treatment of opportunistic infection with these species. *S. paucimobilis* has an innate resistance to colistin, which is a reserve antibiotic. However, there are discussions about an adequate method for testing the polymyxin B/colistin and the critical breakpoints. CLSI recommended “intermediate” or “resistant” categories do not fit the EUCAST categories (EUCAST—Colistin Breakpoints—guidance document 2021). EUCAST guidelines recommend the micro-broth dilution method to detect the resistance to polymixinB/colistin and use colistin sulphate. Although polymixinB/colistin are reserve antibiotics that are not recommended in monotherapy, the emerging of multi-drug resistant species that exhibit the resistance to colistin need adequate interpretative guidelines [17,29,99,100]. A method suitable for the routine testing of polymixinB/colistin susceptibility is of great interest and there are studies that propose alternatives to the microbroth dilution method [101]. 

## 5. Conclusions

We isolated two Gram-negative bacilli, *S. paucimobilis* and *H. alvei*, from cave samples, which were identified by conventional bacteriological methods in order to have comparable outcomes with clinical reports. We sought to review the clinical reports with the environmental species. The environmental species should not be considered contaminants without a thorough analysis. 

Human infections with *S. paucimobilis* and *H. alvei* are rare and reported mostly in debilitated patients with underlying diseases. However, our review that included 49 references and 447 cases stressed that *S. paucimobilis* and *H. alvei* were isolated from immune-deficient and immune-competent hosts and that the source of infections is not easily determined. A deep view of the opportunistic species in their natural habitat could advance the understanding of the infections mainly in immune-competent hosts. 

## Figures and Tables

**Table 1:** The bacterial strains were isolated from cave samples.

Sample Code	Cave	Bacterial Strain	Probability (%) *
PTDF1	Topolniţa	*Aerococcus viridans*	88
PLDF1	Apă din Valea Leșului	*Bacillus cereus/thuringiensis/mycoides*	89
PFDF5	Ferice	*Rhodococcus coprophilus/erythropolis/globerulus*	90
PFDF3	Ferice	*Bacillus cereus/thuringiensis/mycoides*	85
PCDF1	Cloşani	***Sphingomonas paucimobilis*** *Geobacillus thermoleovorans*	**94**
			92
PTSDF2	Tăuşoare	***Hafnia alvei*** *Corynebacterium afermentans*	**86**
			90
PMDF11	Muierilor	*Geobacillus toebii*	91
PMDF11A	Muierilor	*Bacillus smithii*	88
PMDF11B	Muierilor	*Bacillus cereus/thuringiensis/mycoides*	86
PMOS1	Muierilor	***Sphingomonas paucimobilis*** *Bacillus smithii*	**93**
			97
PMOS2	Muierilor	*Geobacillus toebi*	95

* According to Vitek2 identification.

**Table 2:** The antibiotic resistance phenotype of the *S. paucimobilis* and *H. alvei* strains.

Antibiotic	*S. paucimobilis* PCDF1			*S. paucimobilis* PMOS1			*H. alvei* PTSDF2		
	MIC ^1^	CLSI ^2^	EUCAST ^8^	MIC	CLSI ^2^	EUCAST ^8^	MIC	CLSI	EUCAST ^8^
Ticarcillin	32		I	<=8	S	S	<=8	-	S ^5^
Ticarcillin/Clavulanic Acid	16	S	S	<=8	-	S	<=8	S	S
Piperacillin	32	I ^6^	I	<=4	S	S	<=4	S	S
Piperacillin/Tazobactam	16	S	S	<=4	S	S	8	S	S
Ceftazidime	>=64	R	R	2	S	S	32	R ^7^	R
Cefepime	>=64	R	R	<=1	S	S	<=1	S	S
Aztreonam	>=64	-	(-)	16	-	I	8		R
Imipenem	1	S	S	1	S		0.5	S	S
Meropenem	<=0.25	S	S	0.5	S		4	R	I
Amikacin	<=2	S	S	<=2	S		<=2	S	S
Gentamicin	<=1	S	IE ^3^	<=1	IE ^3^		<=1	S	S
Tobramycin	<=1	S	S	<=1	S		<=1	S	S
Ciprofloxacin	<=0.25	S	I	2	R		<=0.25	S	S
Pefloxcin	-	-	-	-	-	-	-	-	-
Minocycline	<=1	S	S	<=1	S		8	I	-
Colistin ^4^	>=16	R	R ^9^	8	R	R ^9^	>=16	R	R ^9^
Rifampicin	-	-	-	-	-	-	-	-	-
Trimethoprim/Sulfamethoxazole	<=20	S	S	<=20	S		<=20	S	S

^1^ MIC (minimal inhibitory concentration); ^2^ the interpretation according to CLSI guideline for the category “Other Non-*Enterobacterales*” with the exception of Colistin that was categorized according to CLSI guideline for *Acinetobacter baumanii*; ^3^ IE (Insufficient Evidence that the species is a good target for therapy); ^4^ Note: increase of breakpoint from 2 to 4 mg/L is already approved (see: Colistin Breakpoints—guidance document 2021); ^5^ S (Susceptible); ^6^ I (Intermediate or Susceptible, increased exposure according to the new EUCAST definition); ^7^ R (Resistant), ^8^ according to MIC Interpretation Guideline of EUCAST for the non-fermentative Gram-negative *Acinetobacter baumanii*; ^9^ the EUCAST interpretation is for polymixin B.

**Table 3:** The overall comparison of the infections with *S. paucimobilis* and *H. alvei*.

Type of Infections (*n* = 447)	*S. paucimobilis* (*n* = 262) *n* (%)	*H. alvei* (*n* = 185) *n* (%)
BSI	116 (44.27)	20 (10.81)
UTI	4 (1.52)	121 (65.41)
respiratory tract infections	17 (6.49)	11 (5.94)
bone or soft-tissue infections	18 (6.87)	7 (3.78)
Intra-abdominal infections	15 (5.72)	25 (13.51)
Head and neck infections	20 (7.63)	0
Ocular infections	17 (6.49)	0
Cardiovascular infections	3 (1.15)	0
Other types of infections	52 (19.85)	1 (0.54)
*t*(14) = 0.49, *p* = 0.32		

**Table 4:** The acquired source of BSIs.

The Acquired Source (*n* = 136)	SP	HA
n (%)	*n* = 116 (%)	*n* = 20 (%)
Hospital 50 (36.76)	50 (43.10)	-
Community 43 (31.62)	43 (37.06)	-
Not specified * 43 (31.62)	23 (19.82)	20 (100)

SP (*S. paucimobilis*); HA (*H. alvei*); * included the cases when not clearly specified the acquired source.

**Table 5:** The underlying conditions of the patients with BSIs.

The Underlying Conditions (*n* = 136)	SP	HA
*n* (%)	*n* = 116 (%)	*n* = 20 (%)
malignancy 45 (33.08)	44 (37.93)	1 (5)
diabetes mellitus 15 (11.02)	15 (12.93)	-
bacteremia associated with contaminated iv fentanyl 6 (4.41)	6 (5.17)	-
kidney transplant 6 (4.41)	-	6 (30)
prematurity 5 (3.67)	4 (3.44)	1 ** (5)
surgery 3 (2.21)	3 (2.59)	-
*Streptococcus pyogenes* infections 2 (1.47)	2 (1.72)	-
end-stage renal disease 3 (2.21)	3 (2.59)	
HIV 2 (1.47)	1 (0.86)	1 (5)
neonatal sepsis 2 (1.47)	2 (1.72)	-
burn injury 2 (1.47)	2 (1.72)	-
chronic obstructive pulmonary disease and steroid use 1 (0.74)	1 (0.86)	-
chronic steroid use 1 (0.74)	1 (0.86)	-
chylothorax 1 (0.74)	1 (0.86)	-
Down syndrome 1 (0.74)	1 (0.86)	-
duodenal atresia 1 (0.74)	1 (0.86)	-
epilepsy 1 (0.74)	1 (0.86)	-
imperforate anus 1 (0.74)	1 (0.86)	-
liver cirrhosis and alcoholism 1 (0.74)	1 (0.86)	-
liver transplantation 1 (0.74)	-	1 (5)
perforated appendicitis 1 (0.74)	1 (0.86)	-
pulmonary embolization and atrial fibrillation 1 (0.74)	1 (0.86)	-
septic arthritis 1 (0.74)	1 (0.86)	-
urethral stone 1 (0.74)	1 (0.86)	-
unclear 9 (6.62)	3 (2.59)	6 (30)
No* 23 (16.91)	19 (16.38)	4 (20)

SP (*S. paucimobilis*); HA (*H. alvei*) *; No means—no underlying conditions reported; ** prematurity with necrotizing enterocolitis perforation.

**Table 6:** The overall comparison of the pediatric infections with *S. paucimobilis* and *H. alvei* according to age.

Age Range	*S. paucimobilis* (*n* = 62) *n* (%)	*H. alvei* (*n* = 10) *n* (%)
0 day to 1 month	11 (17.19)	2 (20)
1 month to 1 year	10 (15.63)	2 (20)
1 to 3 years	6 (9.38)	1 (10)
3 to 6 years	11 (17.19)	1 (10)
6 to 12 years	14 (21.88)	3 (30)
12 to 18 years	10 (16.12)	1 (10)
*t*(10) = 7.84, *p* < 0.00001		

**Table 7:** The comparisons of the type of pediatric infections with adult infections.

Type of Infections (*n* = 447)	Pediatric (*n* = 72)		Adult (*n* = 375)	
*n* (%)	SP *n* = 62 (%)	HA *n* = 10 (%)	SP *n* = 200 (%)	HA *n* = 175 (%)
**BSI 136 (30.42)**	**55 (88.71)**	**8 (80)**	**61 (30.5)**	**12 (6.85)**
primary bacteremia 69 (15.44)	34 (54.83)	4 (40)	25 (12.5)	6 (3.43)
CR-BSI 19 (4.25)	7 (11.29)	0	12 (6)	0
bacteremia associated with contaminated iv fentanyl 6 (1.34)	0	0	6 (3)	0
urosepsis 10 (2.24)	3 (4.83)	1 (10)	0	6 (3.43)
pneumonia 4 (0.89)	1 (1.61)	0	3 (1.5)	0
meningitis 2 (0.45)	0	1 (10)	1 (0.5)	0
bone or soft tissue infections 5 (1.12)	0	0	5 (2.5)	0
intra-abdominal infections * 6 (1.34)	1 (1.61)	2 (20)	3 (1.5)	0
unspecified 15 (3.35)	9 (14.52)	0	6 (3)	0
**UTI 125 (27.96)**	**0**	**0**	**4 (2)**	**121 (69.14)**
**Respiratory tract infections 28 (6.26)**	**1 (1.61)**	**0**	**16 (8)**	**11 (6.29)**
ventilator-associated pneumonia 1(0.22)	0	0	0	1 (0.57)
empyema 1(0.22)	0	0	1 (0.5)	0
airway inflammation 1(0.22)	1 (1.61)	0	0	0
unspecified 25 (5.6)	0	0	15 (7.5)	10 (5.71)
**Bone or soft tissue infections 25 (5.6)**	**2 (3.23)**	**1 (10)**	**16 (8)**	**6 (3.43)**
cellulitis 2 (0.45)	0	0	2 (1)	0
otomastoiditis 1 (0.22)	1 (1.61)	0	0	0
osteomyelitis and septic arthritis 1 (0.22)	1 (1.61)	0	0	0
septic arthritis 1 (0.22)	0	0	1 (0.5)	0
osteomyelitis 2 (0.45)	0	0	2 (1)	0
deep infection, open fracture 1(0.22)	0	1	0	0
focal myositis 1(0.22)	0	0	1 (0.5)	0
necrotizing fasciitis 1(0.22)	0	0	0	1 (0.57)
unspecified soft tissue infections 15(3.36)	0	0	10 (5)	5 (2.86)
**Intra-abdominal infections 40 (8.94)**	**1 (1.61)**	**1 (10)**	**14 (7)**	**24 (13.71)**
peritoneal dialysis-associated peritonitis 14 (3.13)	1 (1.61)	0	13 (6.5)	0
diarrhea 19 (4.25)	0	1 (10)	0	18 (10.29)
cholecystitis 1(0.22)	0	0	0	1 (0.57)
intra-abdominal abscess and peritoneal dialysis related peritonitis 1 (0.22)	0	0	1 (0.5)	0
unspecified 5 (1.12)	0	0	0	5 (2.86)
**Head and neck infections 20 (4.47)**	**3 (4.84)**	**0**	**17 (8.5)**	**0**
brain abscess 1 (0.22)	1 (1.61)	0	0	0
cervical adenitis 1 (0.22)	1 (1.61)	0	0	0
central nervous system infections 6 (1.34)	1 (1.61)	0	5 (2.5)	0
unspecified 12 (2.68)	0	0	12 (6)	0
**Ocular infections 17 (3.8)**	**0**	**0**	**17 (8.5)**	**0**
endophthalmitis 3 (0.67)	0	0	3 (1.5)	0
neurotrophic keratitis 1 (0.22)	0	0	1 (0.5)	0
ocular contaminations 13 (2.9)	0	0	13 (6.5)	0
**Cardiovascular infections 3 (0.67)**	**0**	**0**	**3 (1.5)**	**0**
cardiac implantable electronic device infection 1 (0.22)	0	0	1 (0.5)	0
endocarditis 1 (0.22)	0	0	1 (0.5)	0
acute phlebitis 1(0.22)	0	0	1 (0.5)	0
**Other types of infections 53 (11.86)**	**0**	**0**	**52 (26)**	**1 (0.57)**
periodontal disease 51 (11.41)	0	0	51 (25.5)	0
bromhidrosis 1 (0.22)	0	0	1 (0.5)	0
unclear (body fluid) 1 (0.22)	0	0	0	1 (0.57)
*t*-test, *p*-value	*t*(16) = 0.95, *p* = 0.18		*t*(16) = 0.19, *p* = 0.42	
*F-*ratio, *p*-value	*F* = 1.61, *p* = 0.21			

IV (intravenous); SP (*S. paucimobilis*); HA (*H. alvei*); * (pyomyoma, biliary tract infection, necrotizing enterocolitis perforation, liver abscess, food-borne sepsis); BSI (bloodstream infection); UTI (urinary tract infection); CR-BSI (catheter-related bloodstream infection); in bold we marked the infection categories

**Table 8:** The comparisons of the underlying conditions associated with the pediatric infections and adult infections.

Reported Underlying Conditions (*n* = 447)	Pediatric (*n* = 72)		Adult (*n* = 375)	
*n* (%)	SP *n* = 62 (%)	HA *n* = 10 (%)	SP *n* = 200 (%)	HA *n* = 175 (%)
Malignancy 50 (11.19)	22 (35.48)	1 (10)	27 (13.5)	0
Chronic heart disease 8 (1.78)	0	0	8 (4)	0
Chronic renal disease 25 (5.59)	2 (3.22)	0	16 (8)	7 (4)
Chronic liver disease 5 (1.12)	0	1 (10)	4 (2)	0
Chronic pulmonary disease 7 (1.57)	0	0	7 (3.5)	0
Congenital malformation 2 (0.45) (duodenal atresia, imperforate anus)	2 (3.22)	0	0	0
Genetic disorders 3 (0.67) (Down syndrome, cystic fibrosis)	2 (3.22)	0	1(0.5)	0
Diabetes mellitus 15 (3.36)	0	0	15 (7.5)	0
Surgery 4 (0.89) (cardiovascular surgery, neurosurgery, cataract extraction)	0	0	3 (1.5)	1 (0.57)
Trauma 7 (1.57) (burn injury, penetrating globe injury, history of fracture, remote foreign body aspiration during the dental procedure)	1 (1.61)	1 (10)	5 (2.5)	0
Prematurity * 6 (1.34)	5 (8.06)	1 (10)	0	0
Multiple conditions ** 8 (1.79)	0	0	8 (4)	0
other infections 11 (2.46) (HIV, *Streptococcus pyogenes*, abdominal infections due to perforated appendicitis, septic arthritis, neonatal sepsis, significant dysbiosis associated COVID-19)	4 (6.45)	1 (10)	5 (2.5)	1 (0.57)
Other conditions 16 (3.58) (red eye, chylothorax, epilepsy, respiratory distress)	1(1.61)	0	15 (7.5)	0
Chronic steroid use or alcohol abuse *** 10 (2.24)	0	0	10 (5)	0
No underlying conditions reported 80 (17.9)	22 (35.48)	5 (50)	1(0.5)	52 (29.71)
Unclear or not specify 190 (42.51)	1(1.61)	0	75 (37.5)	114 (65.14)
*t*-test, *p*-value	*t*(32) = −0.503, *p* = 0.31		*t*(32) = 0.37, *p* = 0.35	
*F-*ratio, *p*-value	*F* = 1.56, *p* = 0.21			

SP (*S. paucimobilis*); HA (*H. alvei*); * one *H. alvei* prematurity with necrotizing enterocolitis perforation; ** there is one case of diabetes mellitus, liver cirrhosis, end-stage renal disease, hepatocellular carcinoma, and one case of diabetes mellitus, colonic tuberculosis, end-stage renal disease mentioned at “Multiple conditions” category; *** there are one case of HIV and drug abuse, one case of malignancy and chronic steroid use, one case of chronic pulmonary disease and steroid use, and one case of chronic liver disease, and alcoholism mentioned at “other infections”, “malignancy”, “chronic pulmonary disease”, “chronic liver disease” category, respectively.

## Data Availability

No new data were created or analyzed in this study. Data sharing is not applicable to this article.
